# Exome Profiling Suggests Combined Effect of Myeloperoxidase, Toll-Like Receptors, and Metallopeptidase in Hidradenitis Suppurativa

**DOI:** 10.3390/biomedicines12112498

**Published:** 2024-10-31

**Authors:** Alessia Azzarà, Ilaria Cassano, Carla Lintas, Fiorella Gurrieri

**Affiliations:** 1 Research Unit of Medical Genetics, Department of Medicine and Surgery, University Campus Bio-Medico of Rome, Via Alvaro del Portillo 21, 00128 Rome, Italy; i.cassano@unicampus.it (I.C.); c.lintas@unicampus.it (C.L.); f.gurrieri@unicampus.it (F.G.); 2 Fondazione Policlinico Universitario Campus Bio-Medico, Via Alvaro del Portillo 200, 00128 Rome, Italy; 3 Operative Research Unit of Medical Genetics, Fondazione Policlinico Universitario Campus Bio-Medico, Via Alvaro del Portillo 200, 00128 Rome, Italy

**Keywords:** hidradenitis suppurativa, exome sequencing, TLR, acne inversa, candidate genes

## Abstract

Background: Hidradenitis suppurativa, also called acne inversa, is a chronic skin inflammatory condition involving hair follicles, sebaceous glands, and apocrine glands. Symptoms can be variable in intensity, ranging from mild to severe. The exact causes of hidradenitis suppurativa are not fully understood, but the etiology is presumed to be multifactorial, encompassing genetics and environmental factors. Methods: Two families presented with hidradenitis suppurativa with an autosomal dominant pattern. We performed whole-exome sequencing in two unrelated patients from the two families. Results: We identified two and three variants in the two families, respectively. Variants involved the *TLR2* and *MPO* genes in the first family and the *MMP2*, *GJB2*, and *TLR4* genes, some of which have already been previously reported as possible candidates for hidradenitis suppurativa. Conclusion: It is very likely that variants in a single gene only rarely cause the condition and that most cases, especially familial hidradenitis suppurativa cases, may more probably take the form of polygenic disorders.

## 1. Introduction

Hidradenitis suppurativa (HS), also known as acne inversa, is a chronic skin inflammatory condition involving hair follicles, sebaceous glands, and apocrine glands. This condition results in the formation of painful skin abscesses especially in areas of the body rich in apocrine sweat glands, such as the armpits, groin, buttocks, and skin folds [1].

Symptoms of HS can be variable in intensity, ranging from mild to severe, and may include inflamed cutaneous nodules, abscesses, and pus-discharging lesions, which develop in axillary, inguinal, gluteal, and perianal body sites. A skin biopsy is usually not required for the diagnosis of HS. HS is clinically diagnosed based on the nature and localization of skin lesions and the course of the disease, in terms of the number of skin lesions and timing [2].

The mechanisms underlying HS are complex and result from follicle instability and occlusion, followed by follicle rupture and an aberrant immune response. This creates a pathogenic cycle of tissue damage (which can also begin with mechanical stresses induced by skin–skin rubbing) and bacterial colonization, leading to immune activation around the terminal follicles and hyperkeratosis. These factors lead to immune activation around the terminal follicles and hyperkeratosis [3,4,5].

A total body skin examination is required to assess the extent and severity of HS. Currently, Physician Global Assessment for HS and the International Hidradenitis Suppurativa Severity Score System (IHS4) are useful tools for severity assessment and help to determine the appropriate medical treatment [6]. Usually, a skin biopsy is not required to diagnose HS. The histopathologic features of HS vary according to the type of alteration and may be helpful in understanding the pathogenic development from early stage to advanced disease. Timely diagnosis and early treatment of HS are critical for managing symptoms and minimizing the formation of new lesions. To date, the treatments used for HS aim to reduce the inflammation of pre-existing lesions and prevent the formation of new lesions but also improve the patient’s quality of life. Depending on the degree of lesion and severity of the disease, pharmacological treatment includes the use of antibiotics, anti-inflammatories, and steroids, and also pulsed light treatments and surgery to drain abscesses and remove scar tissue. In this complex and multifactorial condition, it is certainly essential to perform a proper inspection of the patient’s body and also consider familial predisposition. The exact causes of HS are not fully understood: many different factors, including hormonal, immunology, lifestyle, microbiota, and genetic variants have been presumed to play a role in disease progression [7,8,9]. The condition can be worsened by obesity, smoking, stress, and skin irritation [10].

Due to its genetic heterogeneity and etiological complexity, HS can be classified as either familial or sporadic [11]. Typically, HS presents as sporadic disease, but there are cases in which it manifests as a familial disorder, showing an autosomal dominant inheritance (about 40 percent of familial cases) and a genetically heterogeneous pattern [12].

The study of genetics in HS is very important to better define the genetic contribution to the condition. By using haplotype analysis, the first study in two large four-generation families mapped the 1p21.1–1q25.3 genomic region as a possible HS locus, representing a start point for understanding the molecular mechanisms of this disease [7]. In familial HS, monogenic inheritance is rare (less than 7%), and this trait is often related to mutations of γ-secretase component genes and the Notch pathway or to defects in inflammasome-related genes. Among the component genes of γ-secretase, heterozygous LOF (loss-of-function) mutations in presenilin (*PSEN1*), presenilin enhancer 2 (*PSENEN*), and nicastrin (*NCSTN*) [13,14,15] have been identified both in familial cases and in a few sporadic ones.

Subsequently, whole-exome sequencing (WES) in 11 families with HS not only identified mutations in the Notch pathway but also reported multiple variants of unknown significance that segregated with the disease within these families [16]. This study might support the notion that HS is inherited as a polygenic trait including various genes involved in inflammation/inflammasome, skin microbiome, innate and adaptive immunity, keratinization pathway, or epidermal proliferation and homeostasis [17,18,19,20]. This indicates a clear autoinflammatory etiology in which gene alterations, environment, and lifestyle contribute to the development of the disease.

We investigated two different families (family T and family G) where HS segregated with an autosomal dominant pattern. In order to identify one or more variants present in all affected individuals, we performed whole-exome sequencing in a single member of the family. We identified two variants in the T family and three variants in the G family in different genes, previously reported as possible candidates for HS.

## 2. Materials and Methods

### 2.1. Clinical Features of Patients

Family T, patient II-1T.

**Figure 1:**
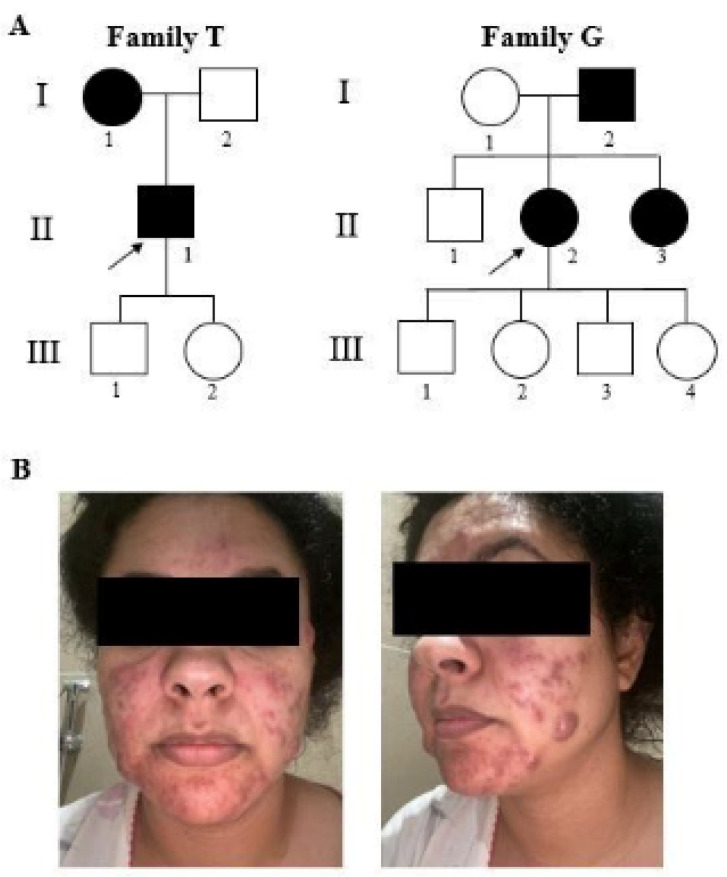
Pedigree of families (**A**) and facial clinical evidence of II-2G (**B**). The black symbols represent affected individuals. Square and circle represents male and female respectively. Arrows indicate the index patients in whom WES was performed.

The patient was 37 y/o (II-1T) at the time of our clinical evaluation (2021). He was referred because of recurrent episodes of subcutaneous abscesses in the face and the rest of the body during the previous 5 years, for which he was constantly under antibiotic treatment, with some relief. When he was 16 y/o, he suffered from juvenile pustular acne, treated with vitamin A and laser therapy. A rheumatological evaluation ruled out possible Bechet’s disease after negative HLAB51 and HLAB27 tests and normal sacroiliac MRI results; a possible systemic autoinflammatory condition was suspected, and a dermatological evaluation raised the suspicion of an infectious condition, indicating the need to carry out a biopsy of the skin lesion, which was not refused by the patient. He reported that his mother also suffered from skin abscesses (Figure 1A). Based on the clinical phenotype and on the family history, a multigene panel for autoinflammatory diseases was analyzed, with normal results. Affected family members also had striking facial lesions, and a comorbidity of acne conglobata was actually present. They did not have any of the known risk factors. We then performed exome analysis on a research basis. No other subjects from this family took part in the study.

Family G, patient II-2G.

The second patient (II-2G) was a 41 y/o female (Figure 1A), who suffered at a young age from marked acne, treated with chemical peeling; since then, she had had recurrent appearances of purulent nodules, strikingly occurring on the face and skin folds, alleviated by contraceptive therapy (Figure 1B). At the age of 40, she underwent an intrauterine device implant, which triggered a new acute diffuse episode. She reported no recurrent fevers, arthritis, uveitis, or canker sores. A diagnosis of hidradenitis suppurativa was made. No risk factors for the disease were reported in this patient. Her father and sister also have recurrent episodes of subcutaneous abscesses.

The mother does not have dermatological issues, except for thick skin and mild late-onset hearing loss. A multigene panel for autoinflammatory suppurative syndromes was negative.

WES was therefore carried out in patient II-2G, and samples were also collected from the parents for segregation analysis.

### 2.2. Whole-Exome Sequencing

Whole-exome sequencing (WES) was performed in II-1T and II-2G patients from two families. Briefly, DNA was isolated from peripheral blood leukocytes, and WES was commissioned to an external service (DanteLabs SRL, Aquila, Abruzzo) aiming for an average coverage of 100× on an Illumina platform. Fastq files were generated for each patient and processed using a customized bioinformatics pipeline through the Galaxy online platform [21]. Initially, paired reads were mapped using BWA [22] (Human GRCh37/hg19), followed by duplicate removal and variant calling using FreeBayes. Annotation was carried out using wAnnovar [23].

We included in our analysis all variants identified within the coding/splicing regions and with a good coverage (read depth cut-off ≥20) and a minor allele frequency (MAF) ≤0.01, and we filtered out synonymous variants.

Variants were then prioritized using Varelect [24], employing a list of genes generated from the analysis along with specific HPO (Human Phenotype Ontology) terms: HP:0031292 “cutaneous abscess”, HP:0011107 “aphthae”, HP:0040154 “hidradenitis suppurativa”, HP:0025615 “abscess”, HP:0002719 “recurrent infections”, and HP:0033428 “autoinflammation”. We also matched the exome data with a list of HS-related genes from the literature. All genes along with their variants were manually inspected by loading all BAM files as a custom track on the UCSC genome browser (https://genome.ucsc.edu/) to confirm their presence. To assess the frequency of each variant, the gnomAD database was queried, and in silico analysis of missense variants was conducted using Varsome and CADD score [25,26]. All bioinformatics analyses were carried out following best practice recommendations [27,28]. Sanger sequencing was performed on each variant that passed the filtering/prioritization procedure using standard methods. The local ethics committee (Institutional Review Board approved n° 04.21) approved the study and it was performed in accordance with the Declaration of Helsinki for Human Rights. All patients enrolled in the study signed a written informed consent.

### 2.3. Direct Sanger Sequencing

To perform segregation analysis, oligonucleotide primers flanking variants were designed using the Primer3 application on the UCSC genome browser. Primer sequences will be provided upon request. Each amplicon was PCR amplified and purified with a 1:1 mixture of Exonuclease III and shrimp alkaline phosphatase at 37 °C for 15 min followed by heat inactivation at 80 °C. After cleaning up, the PCR product was sequenced using a BigDye terminator v3.1 Cycle Sequencing Kit and run on a 3130 Genetic Analyzer (Applied Biosystems, Foster City, CA, USA). The electropherograms were analyzed by the Sequencing Analysis v5.2 software (Applied Biosystems, Foster City, CA, USA).

## 3. Results

After the bioinformatics pipeline, the variants in the 3′ and 5′ UTR and intronic region, synonymous variants, and low-quality and low-coverage (<20X) variants located in non-coding regions were filtered out.

Bioinformatic analysis on exome data for CNV detection was negative.

In a first analysis, we wanted to know if there were variants (to any MAF) in genes that had already been reported in the literature in relation to HS, and afterwards we evaluated rare variants. We then used the VarElect analysis to identify genes prioritized based on keywords related to the conditions. In both patients II-1T and II-2G, about 700 genes with 1000 variant were first matched with genes found to be related to HPO. We prioritized using VarElect, and each variant was manually inspected on the BAM file (loaded on the UCSC genome browser).

The variants in the top 20 genes with the highest score from this analysis were confirmed using Sanger sequencing and were segregated in the family, where it was possible.

Each variant was individually checked, on the basis of MAF in the gnomAD database (https://gnomad.broadinstitute.org/), and evaluated with respect to its global functional effect (“damaging” or “tolerated”) using different in silico tools: CADD score (up to 15) Mutation Taster, SIFT, Polyphen and GERP.

First, we compared variants found in both patients in order to identify possible variants and/or genes shared between the two individuals. This analysis was negative. Based on the MAF and the CADD score, we selected two variants in II-1T and three variants in II-2G (Table 1).

In patient II-1T, we identified two missense variants in the *TLR2* and *MPO* genes: p.(Pro631His) and p.(Ala332Val), respectively. The heterozygous substitution in the *TLR2* gene (toll-like receptor 2) is a C>A change at position 1892 of the coding sequence leading to a p.(Pro631His) substitution at the protein level. The variant has a frequency of 2.8% in gnomAD with a CADD of 25.2. Variants in this gene contribute to susceptibility to leprosy, mycobacterium tuberculosis, and colorectal cancer (OMIM #246300 #607948 #114500).

The variant in the gene *MPO* (myeloperoxidase), c.955G>A, determines the substitution at the protein level of an alanine with a valine in position 332; it has an allele frequency of 1.2% in the gnomAD database. This variant is reported in the ClinVar database (accessed on 18 June 2024, https://www.ncbi.nlm.nih.gov/clinvar/) with conflicting interpretations of pathogenicity (accession number: VCV000003630.15) in relation to a condition known as “Myeloperoxidase deficiency” (OMIM# 254600), an autosomal recessive disease. It was not possible to perform segregation analysis of the variants of II-1T in family T due to low family compliance, although the patient reported recurrent abscesses in his mother, indicating a dominant transmission.

In II-2G patient, we identified three variants: two missense substitutions in the *MMP2* and *GJB2* genes, and one nonsense variant in the *TLR4* gene.

The variant in the gene *MMP2* (matrix metallopeptidase 2), is a T>C substitution at position 1627 of the coding sequence, results in a p.(Tyr543His) at the protein level. This variant with a MAF of 0.010% and a high CADD (score of 30) is reported in the ClinVar database as a variant of uncertain clinical significance (accession number: VCV000885202.8) in relation to “Multicentric osteolysis, nodulosis, and arthropathy” (OMIM # 259600), a recessive condition.

The second missense variant in the gene *GJB2* (gap junction protein beta 2), is a C>T substitution at position 478 of the coding sequence and results in p.(Gly160Ser); it has a frequency of 0.060 in the gnomAD database. This variant is reported in the ClinVar database with conflicting interpretations of pathogenicity (Accession: VCV000044755.60) in homozygous or compound heterozygous states in relation to autosomal recessive deafness (OMIM #220290). Two submissions classify this variant in heterozygosity as a variant of uncertain significance in relation to a condition known as “Keratitis-ichthyosis-deafness syndrome” (OMIM # 148210), which has been reported in relation to some cases of suppurative hidradenitis.

The variant in the *TLR4* gene (toll-like receptor 4) introduces a premature stop codon at position 731. This variant (frequency of 0.0046 in gnomAD) was classified as disease-causing, with a high CADD (score of 40). The *TLR4* gene has a probability of being loss-of-function mutation intolerant (pLI = 0; LOEUF = 0.858). In addition, we performed cDNA analysis to evaluate the eventual transcript degradation of *TLR4* due to nonsense mediated decay activation and the possibility of a loss-of-function effect. After direct Sanger sequencing, we found the presence of both alleles, wild-type and mutated.

In this family, an analysis of the segregation of the variants in the parents of II-2G was carried out: the variants in the *MMP2* and *TLR4* genes were inherited from her father and the variant in the *GJB2* gene from her mother.

## 4. Discussion

In this report, we describe two subjects, II-1T and II-2G, from two different families, who had conditions involving an immune/inflammatory spectrum with different severity but overlapping characteristics. In both cases, after filtering variants obtained by WES in candidate and potentially related genes, we identified multiple variants that might play a synergic role in the clinical manifestations of the patients.

In the T family, subject II-1T predominantly presented with skin-type manifestations in the form of multiple abscesses and lesions in different body sites. Using exome analysis, two variants with a MAF > 1% were identified in two genes recorded in the literature and in the ClinVar database. The p.(Pro631His) missense variant in the *TLR2* gene is reported in the literature in association with an increased susceptibility to conditions known as “Complicated skin and skin structure infections” (cSSSIs), whose clinical characteristic is compatible with the skin manifestations of II-1T [29]. Also, an increased statistically significant expression of TLR2, both at the mRNA and protein levels, was found in chronic inflamed acne inversa lesions compared with normal skin controls [30]. In support of the PolyPhen algorithm tool that predicts the variant Pro631His with damaging effect, there are reports in the literature about the functional characterization of genetic variants in the TLR1-2-6 family, studied by transfection of various TLR constructs in HEK 293T cells containing a NF-κB luciferase reporter construct. Transfection of the TLR2 p.Pro631His led to decreased NF-κB activation (about 30%), compared with the wild-type variant. In addition, it was demonstrated that the presence of the *TLR2* p.Pro631His variant decreases the internalization of the TLR2 complex from the plasma membrane after recognition of its ligand with a subsequently dominant negative effect [31].

The second variant that was found in the *MPO* gene has also been reported in association with “neutrophil accumulation and pustular skin disease” [32]. This gene encodes myeloperoxidase, an essential component of azurophil granules of leukocytes and monocytes. In response to stimulation, MPO is activated with potent antimicrobial oxidizing abilities. MPO deficiency is an autosomal recessive disorder, and most patients are compound heterozygotes. The main clinical characteristic appears to be the common biochemical defect of neutrophils, but other studies have reported an increased susceptibility to infections. In fact, variants of MPO are associated with increased neutrophil accumulation and pustular skin disease [33]. Marchetti et al. (2004) identified our same missense variant p.(Ala332Val) in the *MPO* gene in a male Italian patient with partial myeloperoxidase deficiency, i.e., with neutrophil peroxidase activity [34]. Thus, it is tempting to speculate that the manifestations of MPO variants may be influenced by the genetic background.

It is likely that both variants display a combined effect and, therefore, the condition of II-1T is the result of a synergistic effect in an oligogenic context.

Similarly, we can comment on the identification of the variants in the second patient II-2G. In this patient, segregation analysis in the parents helped in establishing the different phenotype correlations. The mother presented with skin thickening as well as mild, late-onset deafness. The father, as well as the sister, (whose DNA was not available), also showed recurrent episodes of subcutaneous abscesses.

Exome analysis of II-2G revealed three variants: one variant inherited from the mother (in the *GJB2* gene) and two variants inherited from the father (in the *MMP2* and *TLR4* genes).

The variant inherited from the mother is p.(Gly160Ser) in the *GJB2* gene. This gene encodes the gap junction protein connexin 26 (Cx26), which is associated with autosomal recessive deafness (OMIM #220290). GJB2 also regulates the epidermis and hair-follicle keratinization. Heterozygous variants in the GJB2 gene also cause a condition known as “Keratitis-ichthyosis-deafness syndrome” (OMIM #148210), an inherited ectodermal disorder. Variants in this gene have been found in patients with syndromic HS forms by WES, suggesting an altered keratinization pathway in the pathogenesis [35,36,37,38].

The variant in *GJB2* could play a role in some of the clinical aspects shared with the mother, including hypoacusia, presenting variable expressivity.

The first variant inherited from her father is the missense substitution in the *MMP-2* (matrix metalloproteinase-2) gene that is associated with “Multicentric osteolysis, nodulosis, and arthropathy” (OMIM # 259600), caused by homozygous variants.

Some studies have demonstrated the involvement of metallopeptidases, to which *MMP-2* belongs, in the inflammatory process that plays a key role in HS pathogenesis. An overexpression of MMP-2 has been observed in keratinocytes, fibroblasts, and inflammatory cells in dermis analysed in HS-affected skin [39,40]. This could indicate that intrinsic skin components may represent a critical stage that takes part or is involved in the progression of the disease.

The second variant inherited from the father is located in another gene belonging to the TLR family. In this case, it is a nonsense variant located in the *TLR4* gene. Toll-like receptor proteins, such as TLR4, play key roles in the activation of the innate immune response. Although some studies have failed to demonstrate the involvement of variants in TLR4 in determining a certain susceptibility toward hidradenitis suppurativa [41], given the in silico prediction tool scores (CADD score 36) and the role of the TLR4-encoded protein, it is possible to hypothesize that p.(Arg731Ter) may act as a “minor factor” in the II-2G phenotype. Since there was no evidence of nonsense-mediated RNA decay, we might expect that an interference with the product of the wild-type allele with dominant negative effect occurs. Given the evidence from the exome analysis, we hypothesize that both clinical pictures may be the result of a synergistic effect in an oligogenic context.

The etiology of HS is multifactorial, encompassing genetic and environmental factors (i.e., lifestyle, hormonal status, and microbiota) that contribute to the complexity of clinical manifestation. In this context, it is crucial to accurately characterize patients clinically and in terms of their personal and family history.

Since our study relates to two individuals in different families, our results can be helpful and support other laboratories and clinicians in the genetic diagnosis of patients with comparable clinical manifestations by prompting a consideration of these candidate genes.

Certainly, it is necessary to conduct the same study on other families and/or a larger cohort of both sporadic and familial cases in order to strengthen the evidence for the possible involvement of these candidate genes in the disease.

## 5. Conclusions

Through WES, this study contributes to the understanding of the polygenic nature of HS, which points to the inflammation pathway as crucial in the pathogenesis of the disease. It is very likely that pathogenic variants in a single gene only rarely cause HS, and that most cases of HS, especially familial HS cases, may more likely take the form of polygenic disorders.

Future research into the molecular basis of HS, together with functional validation of the identified pathogenic variants, will be important to facilitate the development of targeted treatments for this condition.

## Figures and Tables

**Table 1:** List of the variants identified in two affected individuals with the allelic frequency and in silico evaluation.

	Gene	Position †	RefSeq Gene and HGVS Nomenclature ††	MAF	CADD	Mutation Taster	SIFT	Polyphen	GERP
II-1T	TLR2	chr4:154625951	NM_001318789: c.1892C>A:p.(Pro631His)	7944/282,314	25.2	Uncertain	deleterious	Probably damaging	5.65
	MPO	chr17:56355397	NM_000250: c.995G>A:p.(Ala332Val)	3566/282,336	23.9	Benign moderate	deleterious	Benign	5.32
II-2G	MMP2	chr16:55532218	NM_004530: c.1627T>C p.(Tyr543His)	31/282,540	30	Uncertain	deleterious	Probably damaging	6.07
	GJB2	chr13:20763243	NM_004004: c.478C>T p.(Gly160Ser)	170/282,496	25.2	Uncertain	tolerated	Probably damaging	5.47
	TLR4	chr9:120476597	NM_138554: c.2191C>T p.(Arg731Ter)	13/277,454	40	Disease causing	na	na	6.03

†: Human GRChr37/hg19; ††: Human Genome Variation Society; na: not available.

## Data Availability

Data are contained within the article.
